# Sarcoma nomograms: a light over the darkness

**DOI:** 10.18632/oncoscience.341

**Published:** 2017-03-16

**Authors:** Dario Callegaro, Rosalba Miceli, Alessandro Gronchi

**Affiliations:** Department of Surgery, Sarcoma Service, Fondazione IRCCS Istituto Nazionale dei Tumori, 20133 Milan, Italy

**Keywords:** sarcoma, prognosis, nomogram, staging system

Staging systems for cancer patients are at a turning point. In the 8th edition of the American Joint Committee on Cancer/Union for International Cancer Control (AJCC/UICC) Staging Manual the categorization of patients into prognostic groups is being complemented by nomograms.[1] These instruments aim to compute a personalized probability that a certain event will occur in the future according to specific patient and tumor characteristics.

If we put this transformation into context, we can see that it goes hand in hand with the emergence of precision and patient-centered medicine. On the one hand, allocating the patient into prognostic groups do not adequately inform the actual individual prognosis. On the other hand, prognostic estimates need to be as accurate as possible, considering that the patient is the main actor in the cure process and physician's perspectives exert a great influence on patient decisions.

In rare cancers, such as soft tissue sarcoma (STS), the personalized prognosis prediction is all the more a thorny issue.

From a patient perspective, the rarity of the disease may be perceived as an obstacle to an accurate prognostication.[2] From a physician perspective, validated prognostic models for STS patients are scant. Indeed, taking a look at available prognostic models for STS, excluding GIST, no more than 30% of them are externally validated. The process of external validation consists of applying the prognostic model to an external cohort and evaluating model's performance in terms of discrimination and calibration. Successful external validation in independent series with different case-mix (i.e. the distribution of prognostic factors included in the model) indicate that the model could be generalizable to untested setting. [3]

**Figure 1:**
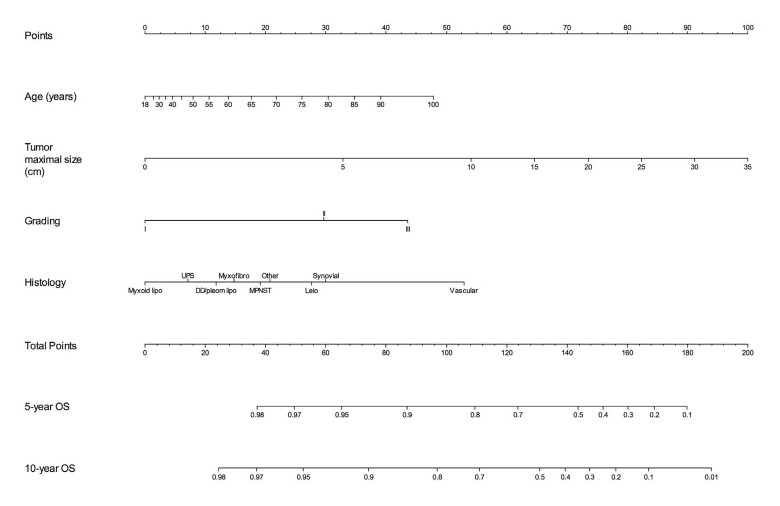
Overall Survival (OS) nomogram for extremity STS patients Figure reprinted with permission: Callegaro, et al. Development and external validation of two nomograms to predict overall survival and occurrence of distant metastases in adults after surgical resection of localised soft-tissue sarcomas of the extremities: a retrospective analysis. Lancet Oncol. 2016 May;17(5):671-80.

In a recent issue of The Lancet Oncology, we presented two new prognostic nomograms to predict overall survival and the metastatic risk at 10 years after surgery in patients with extremity STS.[4] In this paper, three independent series from foreign countries have been used to test the performance of the models proving their generalizability. The nomogram for DM explore the chance of cure of the patient, since the metastatic spread of the disease is almost the only cause of death in this setting. The survival nomogram (Figure 1) predicts the probability to be alive 5 and 10 years after surgery. These nomograms have been also developed in a digital format as a free-app for tablet and smartphone.[5]

The use of these nomograms cannot be separated from the understanding of their creation process and before putting them at the forefront of the clinical practice the physician should answer some questions.

First of all: are these models reliable?

The process of judging the quality of a prognostic model is complex. Checklist of items considered essential for a prediction model have been recently published by the Transparent Reporting of a multivariable prediction model for Individual Prognosis Or Diagnosis (TRIPOD) Initiative (www.tripod-statement.org) and by the AJCC. [6] In other words, a model with good performances in terms of discrimination and calibration is not necessarily reliable. Some methodological issues are essential to be known by the reader and following the stepwise approach of a checklist is useful for this purpose.

The two above-mentioned nomograms for extremity STS both satisfy the quality items of the checklists and achieved good performances at the external validations.

Second: how should we interpret nomogram predictions?

The prognosis calculated by the nomograms are the results of a statistical model. They represent the mean outcome of patients with specific characteristics but the individual variability cannot be caught. For example, if a patient suffers from a severe heart failure, his estimated overall survival will actually probably be lower compared to a healthy patient with all the other covariates being equal. Moreover, nomogram predictions represent the mean outcome of a patient with specific covariates at the time of nomogram development. If a new treatment will change the course of a disease, as it was for example with TKI in GIST, available nomograms would become inaccurate. This means that nomograms need to be updated over time.

Finally: how could the predictions complement the decision-making process?

In the paper by Callegaro et al. we explored the clinical usefulness of these models by means of a decision curve analysis.[4] This method is aimed to assess the net benefit of nomogram-assisted decisions at different threshold probabilities, compared with the net benefit of treat all/treat none strategies.

The next step would be applying these models to the clinical setting. Nomograms could be used to define inclusion criteria for clinical trials, to create nomogram-based therapeutic algorithm or to help identifying the proper follow-up plan.

In a disease, such as STS, where the multidisciplinary discussion of the single case remains the cornerstone of treatment, the application of nomograms to the clinical setting offers the chance to rely the decision- making process on an objective individualized prognostic prediction.

Whether such an approach could be useful for patients is a totally unexplored field but it has the potential to become a new starting point for dealing with this complex family of tumors.
